# Determining an effective short term COVID-19 prediction model in ASEAN countries

**DOI:** 10.1038/s41598-022-08486-5

**Published:** 2022-03-24

**Authors:** Omar Sharif, Md Zobaer Hasan, Azizur Rahman

**Affiliations:** 1 Universal College Bangladesh (Monash College), Dhaka, Bangladesh; 2 School of Science, Monash University Malaysia, Bandar Sunway, Selangor D. E. Malaysia; 3 School of Computing, Mathematics and Engineering, Charles Sturt University, Wagga Wagga, Australia

**Keywords:** Infectious diseases, Statistics

## Abstract

The challenge of accurately short-term forecasting demand is due to model selection and the nature of data trends. In this study, the prediction model was determined based on data patterns (trend data without seasonality) and the accuracy of prediction measurement. The cumulative number of COVID-19 affected people in some ASEAN countries had been collected from the Worldometers database. Three models [Holt’s method, Wright’s modified Holt’s method, and unreplicated linear functional relationship model (ULFR)] had been utilized to identify an efficient model for short-time prediction. Moreover, different smoothing parameters had been tested to find the best combination of the smoothing parameter. Nevertheless, using the day-to-day reported cumulative case data and 3-days and 7-days in advance forecasts of cumulative data. As there was no missing data, Holt’s method and Wright’s modified Holt’s method showed the same result. The text-only result corresponds to the consequences of the models discussed here, where the smoothing parameters (SP) were roughly estimated as a function of forecasting the number of affected people due to COVID-19. Additionally, the different combinations of SP showed diverse, accurate prediction results depending on data volume. Only 1-day forecasting illustrated the most efficient prediction days (1 day, 3 days, 7 days), which was validated by the Nash–Sutcliffe efficiency (NSE) model. The study also validated that ULFR was an efficient forecasting model for the efficient model identifying. Moreover, as a substitute for the traditional R-squared, the study applied NSE and R-squared (ULFR) for model selection. Finally, the result depicted that the prediction ability of ULFR was superior to Holt’s when it is compared to the actual data.

## Introduction

The widespread coronavirus disease (COVID-19) began in China (Hubei Province) in December 2019. Multiple phases of the pattern of the COVID-19 disease continue to be the source of infections in numerous countries, frightening to turn out to be a long-term epidemic. The main reason for spreading this virus is that it was not detected early. Researchers have already begun investigating how the pandemic affects global macroeconomic trends^1^. However, essential to predict how much the virus is spreading rather than measuring global financial loss.

A former study has shown that biological growth models, especially the sub-epidemic growth model, can reveal the empirical shapes of past epidemics that support producing short-term forecasts of an epidemic model in real-time. These epidemic models are beneficial when the collected data are limited for bringing any ideal information^2,3^. In addition, the short period of real-time forecasts created from such models could support preparing to allocate the necessary resources, which are crucial to bringing the epidemic under control.

It is known that each communicable disease epidemic shows specific patterns. Seasonal changes and an adaptation of the virus over time are critical reasons for outbreaks showing different patterns in a country or region. Showing different patterns is typically happening at a different scale level concerning time. Generally, pandemic patterns occur in non-linear shapes. Therefore, this characteristic inspires us to create a model to identify such non-linear lively fluctuations^4^. Moreover, the transmission of such transmittable diseases could be defined by analysing the pattern of these non-linear systems.

The main objective of this research is to determine an effective short–term prediction model by utilizing the cumulative number of COVID-19 infected people in four ASEAN (Association of Southeast Asian Nations) countries, i.e., Thailand, Philippines, Singapore, and Indonesia. To the best of our knowledge, no literature has analysed ASEAN countries’ to predict short term COVID-19 cases, mainly predicting 1 day. Typically, short-term reliable prediction can assist decision-makers in managing the pandemic efficiently in real time and mitigate any upcoming severe risks. Besides, the study focuses on the four countries for the following motives. In the first place, ASEAN countries, especially these four countries, contribute to a vast global economy. Secondly, ASEAN countries' COVID-19 data is available in Worldometers. Therefore, it is crucial to understand how to predict the COVID-19 spreading rate at some ASEAN countries and take effective measures to control the pandemic. Hence, this paper principally uses three dynamic models to generate 1-day ahead or multiple day’s ahead forecasts of the cumulative reported cases of COVID-19 by addressing any seasonal variations. Firstly, the prediction model, Holt’s, and prediction parameters are selected. Secondly, it employs Wright modified Holt’s method for forecasting irregularity in time spacing. Thirdly, an unreplicated linear functional relationship model (ULFR) is applied to find a more justified forecasting value of dependent and independent variables. This ULFR model is a newly used technique in such a type of prediction analysis. Last but not least, the prediction Holt’s model was selected based on data patterns (trend data without seasonality) and the accuracy of prediction measurement with different statistical methods. Particularly, instead of using only the traditional R-squared, this study also used the nash–sutcliffe efficiency (NSE), model efficiency factor (MEF) and the R-squared value of ULFR for model selection.

## Literature review

Since the outbreak in Wuhan, several worldwide scholars and researchers have reported estimations and predictions for the COVID-19 epidemic in journal publications or on websites^5–7^. Among the studies, many modelling results have shown a wide range of variations depending on data availability^8^: estimated basic reproduction number varies from 2 to 6, peak time estimated from mid-February to late March, and the total number of infected people ranges from 50,000 to millions. An important question is now, even though predictions are made using transmission models based on either the Susceptible-Infectious-Removed (SIR) or Susceptible-Exposed-Infectious-Removed (SEIR) framework, why is there such a wide variation in model predictions?^9^. The literature review shows that the primary reason for such variation is selecting a small number of data or selecting parameters without justification^10^.

Recently applied various models for measuring spreading and forecasting COVID-19 such as Ace-Mod Australian Census-based Epidemic Model (Ace-Mod), SIDR, Fuzzy Clustering, SEIR and DASS-21^11–13^. Particularly, Roosa et al.^13^ generate forecasts in China using the Richards growth model, a sub-epidemic wave model, and a generalized logistic growth model. Following models have been previously used to forecast outbreaks due to different infectious diseases. Forecasts finding from each model suggest the outbreaks may be nearing extinction in Guangdong and Zhejiang.

Fokas et al.^14^ applied a computational approach to computing the impact on the number of deaths globally and Paul et al.^15^ predict the disease burden with special emphasis on south Asian countries (India, Bangladesh, and Pakistan) by the SEIR method. Hasan and Siddik^16^ Examine the correlation between daily and total COVID-19 cases in Bangladesh by linear relationship model. Petropoulos and Makridakis^17^ applied exponential smoothing models to predict the continuation of the COVID-19 by analyzing confirmed cases, deaths, and recoveries of several people. Moreover, Md Hasinur Rahaman Khan and Ahmed Hossain^18^ used the Infection Trajectory-Pathway Strategy (ITPS) model to analyze the COVID-19 outbreak situations and predict infections and deaths case from temporal data of confirmed and death cases in Bangladesh. Besides, Fanelli and Piazza^11^ applied the SIDR model to analyze and forecast COVID-19 spreading in China, Italy, France with a concentration in the variables (Susceptible, Infected, Recovered, Dead, Scheme). Besides, Zhou et al.^19^ focused on deep neural network(DNN) and convolutional neural network (CNN) model for behavioural analysis based on heterogeneous health data generated in social media. Furthermore, Mahmoudi et al.^20^ studied the relationship between the spread of Covid-19 and population size by applying the Fuzzy clustering model in USA, Spain, Italy, Germany, UK, France, and Iran. Even though a few researchers used regression models and Genetic programming for short-term prediction of COVD-19 cases in the different regions due to the small number of data or parameter selection problems, many of those models’ outcomes have shown a wide range of dissimilarities^10,17^. Notably, some studies^13–17,21^ have used various mathematical models to determine the spread of the disease, predict the number of incidences, deal with healthcare missing data, health care facilities in tackling COVID-19 spread. In addition, some researcher uses the SEIR model, Genetic Programming and Regression model for short -term prediction^22–24^. Moreover, a dynamic model, a generalized logistic growth model, the Richards growth model, and a sub-epidemic wave model were used to generate 5-day and 10-day ahead forecasts of the cumulative reported cases in the provinces of Guangdong and Zhejiang, China^13^. Consequently, selecting the appropriate model and parameter value is key for the forecasting model. Besides, many models are available in the literature to model infectious diseases. A few models have been used primarily for the countries where the number of cases is very high, like China, Italy, Spain, the UK, Germany and the USA. Still, no literature has analysed ASEAN countries’ to predict short term COVID-19 cases mainly predicting for 1 day.

## Methodology

In forecasting, the popular methods are the average approach, Naïve approach, Drift method, Seasonal naïve approach, Time series methods, Econometric forecasting methods, artificial neural networks. In economics, the widely applied methods fall in Time series methods and Econometric forecasting methods. However, in health science, rarely forecasting models are applied. Unfortunately, COVID-19 teaches us the importance of prediction results to control the spread of the disease. For the model selection, the study first applied the rule of thumb. Then after analyzing data, three models (Holt’s method, Wright’s modified Holt’s method, and unreplicated linear functional relationship model (ULFR)) have been utilized to identify an efficient model for short-time prediction. Afterwards, this study has tested different smoothing parameter by MAPE, MAD, MSE and RMSE to select effective smoothing parameters. Finally, the model is validated by the NSE, MEF, traditional R-squared, and R-squared (ULFR). Therefore, the present study relies on the three mentioned models that are first applied in health science to forecast the number of COVID-19 incidences in four ASEAN countries based on the actual historical data of August 20, 2020, to September 16, 2020.

### Holt’s method

To build Holt's method, first, the exponential smoothing technique was projected in the late 1950s^25^. In addition, the exponential smoothing technique has motivated few of the most practical forecasting approaches. The exponential smoothing method to produce forecasts is defined as weighted averages of former observations. In other words, Holt's is defined as a linear-exponential smoothing. This smoothing model is well known for forecasting data with trends. This model consists of three individual equations that are applied together to create a final forecast result. Among the three equations, the first equation is a smoothing equation that replaces the last period's trend value with the last smoothed value. Besides, the second equation is called the trend equation. The second equation consists of the changes between the last two smoothed values. The final equation consists of level and trend values to find the forecast value. Two parameters are used in Holt’s method are called smoothing parameters. One parameter is used for overall smoothing, and the other one is used for trend smoothing. Therefore, another name of holt’s method is the double exponential smoothing or trend exponential smoothing model^26^. It can be expressed by the following three equations:

1 $${\text{Level}}\;{\text{equation}}\; Y_{t} = \alpha X_{t} + (1 - \alpha )(Y_{t - 1} + Z_{t - 1} )$$

2 $${\text{Trend}}\;{\text{ equation}}\; Z_{t} = \beta (Y_{t} - Y_{t - 1} ) + (1 - \beta )Z_{t - 1}$$

3 $${\text{Forecast}}\;{\text{ equation}}\;F_{t,k} = Y_{t} + KZ_{t}$$

Herein the smoothing constant and variables are defined below: $Y_{t}$ Estimate of the level of the series at time t, $Z_{t}$ Estimate of the trend (slope) of the series at time t, *α (0* ≤ *α* ≤ *1)* Smoothing parameter for the level, *β (0* ≤ *β* ≤ *1)* Smoothing parameter for the trend, $X_{t}$ Estimate of the period t base level from the current period, $Y_{t - 1} + Z_{t - 1}$ Estimate of the period t base level based on previous data.

To calculate the optimal forecasting of the Eq. (3), the following optimization technique to minimizing the squared error over all data points: $Min\,\,\sum {(y_{t} - Y_{t} - KZ_{i} )^{2} }$.

To calculate $Z_{t}$ the following two quantities are taken as a weighted average:

1. Estimation of a trend from the current period from the upsurge in the smoothed trend between the periods (t-1) and t.
2. $Z_{t - 1}$, which is the previous estimate of the trend.

To start Holt’s method, $Y_{0}$ is an initial estimate of the level and another an initial estimate is called $Z_{0}$ which is used of the trend. Here, $Z_{0}$ equals to previous year’s average increase in the time series and $Y_{0}$ equals to last observation.

### Wright’s modified Holt’s method

Wright^27^ introduced a modification of Holt’s method for the data with irregularity in time spacing. However, modified Holt’s method has some unique characteristics, i.e. it has greater computational efficiency and flexibility in terms of having more smoothing constants. Besides, the modified model has a better performance record with empirical data with missing data or zero data.

The notation and forecasting equation of Wright’s modified Holt’s method is illustrated below:

4 $${\text{One}}\;{\text{ period}}\;{\text{ahead}}\;{\text{forecast}}\;{\text{of}}\;{\text{the}}\;{\text{next}}\;{\text{infected}}\;{\text{number:}}\ f_{n + \,1} \, = \,l_{n} \,\,\, + \,m_{n} (\,t_{n} - \,t_{n - 1} )$$

5 $${\text{Intercept}}\;{\text{ of}}\;{\text{ the}}\;{\text{ trend}}\;{\text{ line}}\;{\text{ at}}\;{\text{ disease}}\;{\text{ infected}}\;{\text{ number}}\;{\text{ n }}:l_{n} \, = \,(1 - v_{n} )[l_{n - 1} \,\,\, + \,m_{n - 1} (\,t_{n} - \,t_{n - 1} )] + v_{n} x_{n}$$

6 $${\text{Slope }}\;{\text{of}}\;{\text{ the}}\;{\text{ trend}}\;{\text{ line}}\;{\text{ at}}\;{\text{ disease}}\;{\text{ infected }}\;{\text{number}}\;{\text{ n}}\;:m_{n} \, = \,(1 - u_{n} )m_{n - 1} + \frac{{u_{n} (l_{n} - l_{n - 1} )}}{{(\,t_{n} - \,t_{n - 1} )}}$$

For the Eqs. (5), (6) the variables are defined with following equations

$$u_{n} \, = \,\frac{{u_{n - 1} }}{{(\,d_{n} + \,u_{n - 1} )}},v_{n} \, = \,\frac{{v_{n - 1} }}{{(\,b_{n} + \,v_{n - 1} )}},d_{n} \, = (1 - \beta )^{{(\,t_{n} - \,t_{n - 1} )}} \,\,\,$$

$$b_{n} \, = (1 - \alpha )^{{(\,t_{n} - \,t_{n - 1} )}} ,u_{0} \, = 1 - (1 - \beta )^{q} ,v_{0} \, = 1 - (1 - \alpha )^{q}$$

The initializing levelling and trending values are denoted by $l_{0} \, = a$, $m_{0} \, = b$. Moreover, Infected size and period at disease infected number n are denoted by $x_{n}$ and $t_{n}$ respectively. Additionally, smoothing constant of intercept and slop are presented by $\tilde{\alpha }\,$ and $\tilde{\beta }$ respectively. To initialized, the following constant a and b sequentially represents intercept of trend line and slope of trend line.

### Unreplicated linear functional relationship model (ULFR)

The linear regression model is a popular model for analysing the dependent and independent variables' relationship. Nevertheless, the relationship between the variables becomes a fuzzy relationship due to unusual fluctuations with defined variables. Moreover, as^28^ mentioned in an article, it is unlikely to measure precisely independent variables in all circumstances.

The relationship model for the two variables is introduced following way by considering the above issues. Assume that X and Y are two linearly related unobservable variables. In the Eq. (7) X and Y are defined as a linearly independent variable and target variable respectively.

7 $$Y_{i} = \beta_{a} + \beta_{f} X_{i}$$

The parameters value $\beta_{a}$, $\beta_{f}$ of the Eq. (7) can be found by least square loss function (minimizing the squared error) over all data points : $Min\,\,\sum {(Y_{i} - \beta_{a} - \beta_{f} X_{i} )^{2} }$.

The linear functional relationship model assigns dependent and independent variables by assuming that both the variables are subject to errors. Let, two variables $X_{i}$ and $Y_{i}$ which correspond to random variables $x_{i}$ and $y_{i}$ that are observed with errors, $d_{i}$ and $e_{i}$ respectively, such that,

8 $$\left. {\begin{array}{*{20}l} {y_{i} = Y_{i} + e_{i} } \hfill \\ {x_{i} = X_{i} + d_{i} } \hfill \\ \end{array} } \right\}\;\;i = 1,2, \ldots ,n\,$$

The following conditions are assumed:

Both of the errors have zero mean: $E( d_{i} ) \, = \, E( e_{i} ) = 0$.

The observed errors have constant but different variance $Var(d_{i} ) = \sigma^{2}_{d} \,,Var(e_{i} ) = \sigma^{2}_{e} \,,\forall i$.

The errors are uncorrelated, i.e., $$\begin{aligned} & Cov(d_{i} ,d_{j} ) = Cov(e_{i} ,e_{j} ) = 0,\,\,\,\,\,i \ne j \\ & Cov(d_{i} ,e_{j} )\, = 0,\,\forall i,j \\ \end{aligned}$$.

Chang et al.^29^ termed the defined Eqs. (7) and (8) as ULFR model. In this model, it is assumed that errors $d_{i}$ and $e_{i}$ are mutually independent and normally distributed random variables.

When the ratio of the error variance is known, that is $\frac{{\sigma^{2}_{e} }}{{\sigma^{2}_{d} }}\, = \,\lambda$ to maximize likelihood estimators of parameters $\beta_{a} ,\beta_{f} ,\sigma^{2}_{d}$ and $\,X_{i\,}$ which are derived by differentiating equation *likelihood function* with respect to $\beta_{a} ,\beta_{f} ,\sigma^{2}_{d} \,,\,X_{i\,}$ respectively and equate the result to zero. Subsequently, the equations are simplified with the maximum likelihood estimators to find the parameters. The parameters and variables i.e. $\beta_{f} ,\beta_{a} ,\,\sigma^{2}_{d} \,$ and $X_{i\,}$ are defined as follows (Chang et al.^29^):

9 $$\hat{\beta }_{a} = \overline{y} \, - \hat{\beta }_{f} \overline{x} \,\,\,$$

10 $$\hat{\beta }_{f} = \frac{{(D_{yy} - \lambda D_{xx} ) + \{ (D_{yy} - \lambda D_{xx} )^{2} + 4\lambda D^{2}_{xy} \}^{\frac{1}{2}} }}{{2D_{xy} }}$$

11 $$\hat{\sigma }^{2}_{d} = \frac{1}{n - 2}\left[ {\sum {(x_{i} - \widehat{X}}_{i} )^{2} + \frac{1}{\lambda }\sum {(y_{i} - \hat{\beta }_{a} - \hat{\beta }_{f} \widehat{{X_{i} }})}^{2} } \right]\,\,\,\,\,$$

12 $$\widehat{{X_{i} }} = \frac{{\lambda x_{i} + \hat{\beta }_{f} (Y_{i} - \hat{\beta }_{a} )}}{{\lambda + \hat{\beta }_{f} }}$$

where the mean of x and y denoted and defined by $\overline{y} = \frac{{\sum {y_{i} } }}{n},\overline{x} = \frac{{\sum {x_{i} } }}{n}$.

And the variable $D_{xy} ,D_{yy} ,D_{xx}$ are defined respectively,

$$D_{xy} = \sum {(x_{i} - \overline{x} )} (y_{i} - \overline{y} ),\,\,D_{yy} = \sum {(y_{i} - \overline{y} )}^{2} ,\,\,D_{xx} = \sum {(x_{i} - \overline{x} )}^{2}$$

Additionally, Coefficient of determination of ULFR ($R^{2}_{f}$) for $\lambda = 1$.

Proportion of variance: $R^{2}_{f} = \frac{{D_{r} }}{{D_{yy} }}\,$ and regression sum of square: $D_{r} = \,\frac{{\hat{\beta }_{f} (D_{yy} - D_{xx} ) + 2\hat{\beta }_{f} D_{xy} }}{{1 + \hat{\beta }^{2}_{f} }}$.

### Model selection

In the model selection process, coefficient of determination, NSE and MEF are applied even though the NSE is nearly identical to the coefficient of determination. The main difference is how it is applied^30^.

### Coefficient of determination

The coefficient of determination $R^{2}$ is express as the squared value of the coefficient of correlation according to the common method of its definition. Conventionally, when a set of data sets $(o_{i} ,p_{i} ,i \, = \, 1, \, 2, \ldots , \, n)$ are obtained, a mathematical model $\hat{p}\, = \,f(o_{1} ,o_{2} \ldots o_{m} )$ could be formed to predict *p* based on the observed values of $o_{j}$,${\text{ j}} = \, 1, \, 2, \ldots ,{\text{ m}}$, where *p* is defined as outcome variable and values of $o_{j}$ are termed as observed variables.

The functional form of R-squared is as follows:

$$R^{2} = \left( {\frac{{\sum\limits_{i = 1}^{n} {(o_{i} - \overline{o}_{i} )(p_{i} - \overline{p}_{i} )} }}{{\sqrt {\sum\limits_{i = 1}^{n} {(o_{i} - \overline{o}_{i} )^{2} } } \sqrt {\sum\limits_{i = 1}^{n} {(p_{i} - \overline{p}_{i} )^{2} } } }}} \right)^{2}$$

### Nash–Sutcliffe efficiency

To avoid the shortfall of R-squared, the study also used the NSE factor presented by^31^ to determine the efficiency of the models:

$$NSE = 1 - \frac{{\sum\limits_{i = 1}^{n} {(o_{i} - p_{i} )^{2} } }}{{\sum\limits_{i = 1}^{n} {(o_{i} - \overline{p}_{i} )^{2} } }}$$

This formula can be applied for linear regression and original data on any model. Besides, NSE values can be negative for the non-linear models. The value of NSE could be between $- \infty$ and 1. Naturally, analysts seek an NSE value close to 1 for the best performance of a model. The negative result of NSE specifies an improper model efficiency. The NSE has an excellent reputation for analysing the efficiency of any model, especially the frequently applied model in hydrology^32,33^. Besides, this study is also introduced the model efficiency factor (MEF) to give a comprehensive explanation of NSE from a different perspective, i.e.

$$MEF\, = \,\sqrt {1 - \,NSE}$$

Any smaller value of MEF shows the validation of the model. Thus, the values of MEF ranges between 0 and 1, where the smallest value of the MEF shows excellent models’ performance. However, the value of MFE zero indicates an error-free model, which is not convincing result^34^.

### Dataset

The data in this research were collected from Worldometers info (https://www.worldometers.info/coronavirus/), provided with confirmed cases in ASEAN countries from 20th August to 16th September 2020. There are several reasons behind collecting data from Worldometer. First, there is no reliable free data source for ASEAN countries except Worldometer. Secondly, Worldometer is accredited as the oldest and most reliable data source by the American Library Association (ALA), Johns Hopkins CSSE, Financial Times, The New York Times and Government of UK. Finally, Worldometer is cited as a source of data in thousands of renowned journal articles. The data set in this study included all the ASEAN countries.

Since, trend data are the pre-requisite for better predicting using Holt’s method^26^, therefore some ASEAN countries, Laos, Vietnam, Brunei, Cambodia, Myanmar were excluded from the study were excluded from this study. Besides, we were unable to get the consistent trend pattern of COVID-19 infected cases for these countries. Even though Wright’s modified Holt’s method can deal with zero values^27^, the number of zero values was almost all for these countries.. The summary statistics of the data set is given in Table 1. In the table, it is seen that total number data is 28 for all the country. However, standard deviation and maximum and minimum value has huge difference because each country has different number of population.

**Table 1:** Summary statistics of COVID-19 infected individuals.

Country	N	Range	Minimum	Maximum	Mean	SD
Thailand	28	101	3389	3490	3429.75	30.349
Philippines	28	95,335	177,579	272,914	226,230.07	27,653.601
Singapore	28	1415	56,099	57,514	56,869.36	413.130
Indonesia	28	81,782	147,211	228,993	184,352.93	25,341.732

## Result and discussion

### Selection of smoothing constant for the prediction model

For obtaining the significant result from Holt’s model, it is imperative to determine the best combination of smoothing constant. Therefore, various models such as Ace- Mod (Australian Census-based Epidemic Model)^35^, neural network-based models^36^ and others have been employed to access the situation. However, though these models are significant, they are not appropriate for trend and seasonal data^25^. Table 2 shows the best combinations of smoothing constants for different days in four ASEAN countries. These findings are validated by MAPE (mean absolute percentage error) value as it is mentioned that the MAPE is one of the most popular measures of forecast accuracy^37^.

**Table 2:** Best combinations of smoothing constants for short term prediction. *Source*: Authors’ calculations.

Day	Thailand (α , β)	Philippine (α , β)	Singapore (α , β)	Indonesia (α , β)
Day 1	(0.2,0.1)	(0.9,0.8)	(0.9,0.7)	(0.3, 0.9)
Day 3	(0.9,0.7)	(0.7,0.7)	(0.7,0.5)	(0.8,0.2)
Day 7	(0.9,0.7)	(0.4,0.9)	(0.6,0.6)	(0.4,0.5)

### Model validation

Several studies on validating the forecasted model of COVID-19 transmission are based on R-squared value only. Nevertheless, our research examined the accuracy and validated our prediction models using R-squared, R-squared (ULFR), NSE, and MEF values**.** From Table 3, it is noticed that the ULFR model predict the total cases (1-day ahead) with better accuracy compared with Holt’s. In addition, the ULFR with a 1-day ahead prediction had the highest accuracy among the three different days’ predictions. Furthermore, it is noticed that Indonesia and Singapore have the highest R-squared and NSE value, which approaches unity. Besides, Indonesia and Singapore show the lowest MEF, which approaches zero. Therefore, to find the forecasting accuracy of the proposed model, this study has been verified by comparing two data sets, i.e., the forecasted data and the reported data. These data sets are applied in the following statistical model to validate the model and maximize the forecasting accuracy. All the statistical evidence shows that ULFR is the best prediction model than Holt’s model. Moreover, in the result and discussion section the modified Holt’s method (Wright’s model) is not included because Holt’s and Wright’s model showing the same result and it is accepted because there was no missing observations in our studied data^38^.

**Table 3:** Results of Statistical analysis for selecting the most efficient model between ULFR and Holt’s. *Source*: Authors’ calculations.

	Thailand			Philippines			Singapore			Indonesia		
	Day 1	Day 3	Day 7	Day 1	Day 3	Day 7	Day 1	Day 3	Day 7	Day 1	Day 3	Day 7
R-squared (Holt’s)	0.9940	0.9930	0.6760	0.9990	0.9900	0.9350	0.9920	0.9540	0.6920	**1.0000**	0.9990	0.9970
R-squared (ULFR)	0.9970	0.9677	0.8331	0.9997	0.9950	0.9595	0.9986	0.9770	0.7621	0.9999	0.9993	0.9983
NSE (Holt’s)	0.9875	0.9257	0.5635	0.9997	0.9999	0.9994	1.0000	**1.0000**	1.0000	0.9999	0.9999	0.9996
NSE (ULFR)	0.9934	0.9959	0.9407	0.9997	1.0000	0.9997	1.0000	**1.0000**	1.0000	0.9999	1.0000	0.9997
MFE (Holt’s)	0.0814	0.2725	0.6606	0.0162	0.0102	0.0251	0.0010	**0.0014**	0.0032	0.0112	0.0073	0.0188
MFE (ULFR)	0.0814	0.0637	0.2435	0.0159	0.0039	0.0180	0.0010	**0.0010**	0.0022	0.0111	0.0057	0.0178

Bold marks indicate significant results at 0.05 level.

### Forecasting by Holt’s Method and ULFR

**Figure 1:**
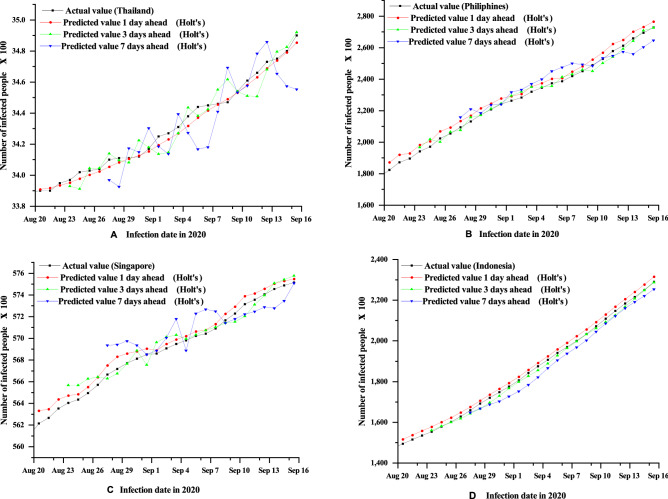
Holt’s method short term (1, 3, 7 days) forecasting of COVID-19 cumulative cases for the four countries Thailand (**A**), Philippines (**B**), Singapore (**C**) and Indonesia (**D**).

**Figure 2:**
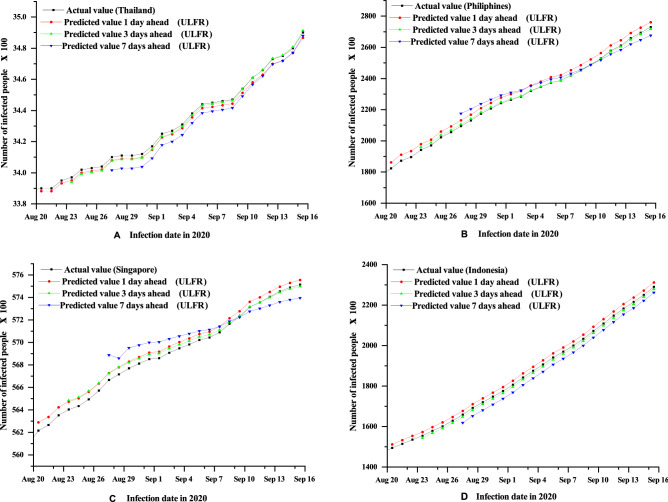
ULFR method short term (1, 3, 7 days) forecasting of COVID-19 cumulative cases for the four countries Thailand (**A**), Philippines (**B**), Singapore (**C**) and Indonesia (**D**).

In this section, Holt’s and ULFR methods have been applied to predict the number of confirmed cases in ASEAN countries, particularly in Thailand, Philippines, Singapore and Indonesia. The goal is to find accurate predictions, which is probably impossible, but to illustrate some general qualitative behaviours which may be observed^39^. Therefore, Figs. 1 and 2 show the cumulative cases of the forecast for 1, 3 and 7 days ahead by Holt’s and ULFR methods respectively. From Fig. 1, it is seen that Holt’s method shows better results for the Philippines and Indonesia for 1, 3 and 7 days. However, the forecasting result is not up to the mark for Thailand and Singapore.

**Figure 3:**
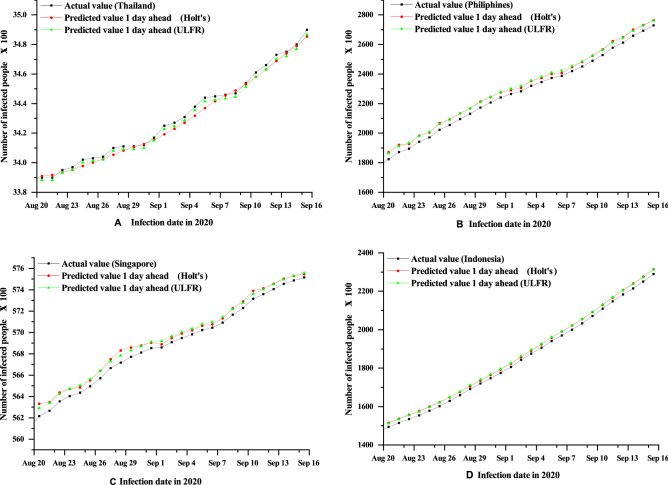
Comparison between Holt’s and ULFR method—1 day ahead forecasting of COVID-19cumulative cases for the four countries Thailand (**A**), Philippines (**B**), Singapore (**C**) and Indonesia (**D**).

**Figure 4:**
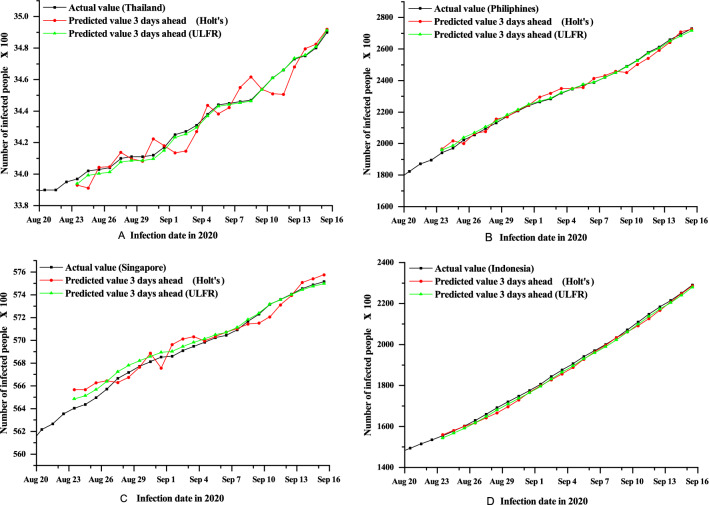
Comparison between Holt’s and ULFR method—3 days ahead forecasting of COVID-19 cumulative cases for the four countries Thailand (**A**), Philippines (**B**), Singapore (**C**) and Indonesia (**D**).

**Figure 5:**
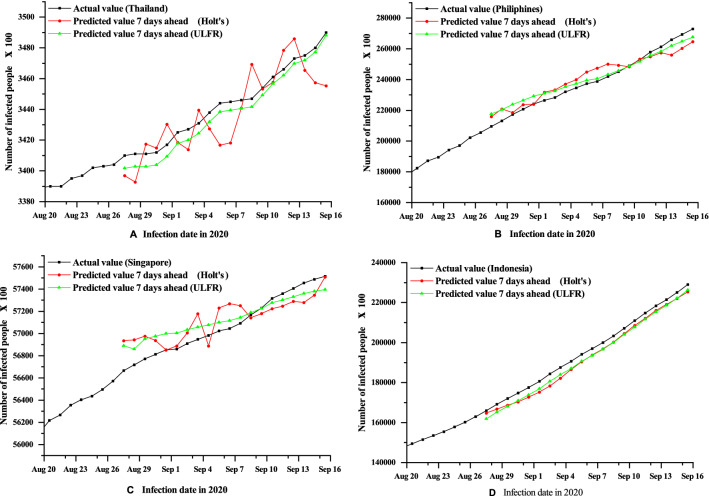
Comparison between Holt’s and ULFR method—7 days ahead forecasting of COVID-19 cumulative cases for the four ASEAN countries Thailand (**A**), Philippines (**B**), Singapore (**C**) and Indonesia (**D**).

On the other hand, ULFR in Fig. 2 shows the descent prediction result for all four countries. Therefore, ULFR gives a better prediction result than Holt’s. The fluctuating case may partially explain that long term prediction is less significant for both models even though ULFR shows significant results than Holt’s. Additionally, it is seen clearly form the Figs. 3, 4 and 5 that short term (1 day) prediction is more accurate than long term prediction. In this case, ULFR shows a perfect prediction result than Holt’s method. There are studies that investigate the increasing rate at Globally or nationally. For example, Rohit et al.^40^ has mentioned that COVID-19 is increasing globally at a rate of 3% to 5% daily. Sharif et al.^10^ commented in their forecasting analysis that the cumulative number of infected cases would be raised to around 500,000 by 2021 in Dhaka division, Bangladesh. However, these studies did not show any justified prediction comparison. But, in our study we have compared original data and predicted data through graphical presentation.

After comparing Figs. 3, 4 and 5 it is clearly visible that day 1 prediction result shows highest accuracy in prediction. Besides, statistical analysis in the Table 3 depicts that highest number of coefficient of determination values of Holt’s and ULFR model for 1 day prediction. It is also observed that Fig. 4 depicts better accuracy than Fig. 5 which means 3 days prediction is better than 7 days prediction. However, in a study on deep learning-based forecasting model for COVID-19 outbreak in Saudi Arabia, Brazil, India, Saudi Arabia, South Africa, Spain, and USA^41^ found that different countries has different trends. But, that study did not examine all the counties’ prediction result with justification. They just showed their accurate result justification by using conventional RMSE, MSE and $R^{2}$ value. However, in our study we have analysed prediction result validation by coefficient of determination, NSE and MFE for both Holt’s and ULFR result which are presented in the Table 3.

Moreover, Roosa et al.^13^ analyzed daily reported cumulative data of COVID-19 cases of China (Guangdong and Zhejiang) generated from13 to 23 February 2020 to forecast only 10-day ahead cumulative case by using a generalized logistic growth model, Richards growth model, and a sub-epidemic wave model. Even though the study mentioned that short term prediction, they did not define short term is how much short. In our study we have used deferent regional data and compared the data for different days. In Figs. 1, 2, 3, 4 and 5 it is clearly visible that Philippines and Indonesia forecasting result showing accurate as all the curves in graph B and D are showing perfectly positive regression. However, prediction result of Thailand and Singapore showed moderately positive regression as the prediction result and original result are fluctuated marginally which is visible in the Figs. 1, 2, 3, 4 and 5 C and D section.

In another study the neuro-fuzzy system was used by Al-qaness et al.^42^ by incorporation with marine predators algorithm to predict the COVID-19 cases in Italy, South Korea, Iran, and USA. A high coefficient of determination was obtained for all the forecasted nations; it was 96.48%, 96.96%, 98.74%, and 98.59% for South Korea, Iran, USA, and Italy respectively. On the other hand, in our study a very high coefficient of determination was obtained for day one and day three ahead prediction for all the counties; it was around 99% for all the four countries (Thailand, Philippines, Singapore and Indonesia). However, worst prediction result was seen for 7 days ahead which is around 67% for Thailand.

## Conclusion

To sum up, this is the first study to predict the number of infected COVID-19 cases in some ASEAN countries using Holt’s and ULFR approaches. Based on our findings, for the short term (1-day prediction), Holt’s and ULFR show better results. However, ULFR shows a significant result for 3 and 7 days too. This study’s outcome could help ASEAN countries’ governments observe the current COVID-19 condition and apply the forecast result to avoid further transmissions. We recommend conducting a follow-up study as many external factors estimate 100% forecast accuracy even though it is impossible to find 100% accurate results. For example, the daily COVID-19 infected cases reported by the government might be lower than the actual number of cases due to delays in publishing test results. Besides, some people would be immune before even screening the COVID-19 test.

There are a few limitations in this study that this study did not include more than 7 days’ prediction due to a small data range. Besides, this study could not have all ASEAN countries due to a lack of balance data. Moreover, this study concentrated only on one factor: the number of confirmed cases. Thus, for future study, the next step would be to apply the Holt’s and ULFR model to forecast optimal parameters such as the total confirmed cases, total deaths, and total recovered cases for more than 7 days ahead.
